# Men’s view on participation in decisions about prostate-specific antigen (PSA) screening: patient and public involvement in development of a survey

**DOI:** 10.1186/s12911-020-1077-4

**Published:** 2020-04-06

**Authors:** Søren Birkeland, Susanne S. Pedersen, Anders K. Haakonsson, Michael J. Barry, Nina Rottmann

**Affiliations:** 1 OPEN, Open Patient data Explorative Network, Department of Clinical Research, University of Southern Denmark, J. B. Winsløws Vej 9 a, 3 Floor, DK-5000 Odense C, Denmark; 2 Department of Psychology, University of Southern Denmark, Campusvej 55, DK-5230 Odense M, Denmark; 3 Department of Cardiology, Odense University Hospital, J. B. Winsløwsvej 4, DK-5000 Odense C, Denmark; 4 Division of General Internal Medicine, Massachusetts General Hospital & Harvard Medical School, 50 Staniford Street, 9th Floor, Boston, MA 02114 USA; 5 REHPA, The Danish Knowledge Centre for Rehabilitation and Palliative Care (Odense University Hospital), Winsløwparken 19, 3. sal, DK-5000 Odense C, Denmark; 6 Department of Clinical Research, University of Southern Denmark, Winsløwparken 19, 3. sal, DK-5000 Odense C, Denmark

**Keywords:** Cancer, Prostate cancer, Health care users’ experiences, User-involvement, Patient and public involvement, Patient engagement, Patient satisfaction, Malpractice litigation, Bioethics, Medical law

## Abstract

**Background:**

Prostate-Specific Antigen (PSA) screening for early detection of prostate cancer (PCa) may prevent some cancer deaths, but also may miss some cancers or lead to unnecessary and potentially harmful treatment. Therefore, involving patients in decision-making about PSA screening is recommended. However, we know little about the attitude of men regarding participation in decisions about PSA screening and how to assess such attitudes. The purpose of this paper is to describe patient and public participation in the development of a national, web-based case vignette survey for studying men’s view on participation in decision-making about PSA screening.

**Methods:**

The project group developed a first draft plan for the survey, its vignettes and choice of measurements. This included multiple vignette variants representing various levels of patient participation in decision-making about PSA screening with different outcomes. Additionally, it included questions on respondents’ satisfaction with imagined courses of health care, their propensity to initiate a malpractice complaint, their own health care experiences, socio-demography, personality, and preferences for control regarding health care decision-making. Following feedback from a workshop with academic peers on the draft plan, a group of 30 adult men was engaged to help develop case vignette versions and questionnaire items by providing feedback on structure, comprehension, response patterns, and time required to complete the survey. Furthermore, a panel of three patients with PCa experience was assembled to assist development through a separate review-and-feedback process.

**Results:**

Based on reviews of survey drafts, the large group made further suggestions about construction of the survey (e.g. clarification and modification of case vignette versions, deletion of items and adjustment of wording, instructions to guide respondents, replacement of technical terms, and optimization of sequence of survey elements). The patient panel ensured fine-tuning of vignette versions and questionnaire items and helped review the internet version of the survey.

**Conclusions:**

Patient and public involvement during various phases of the survey development helped modify and refine survey structure and content. The survey exemplifies a way to measure health care users’ satisfaction with imagined courses of health care and wish to complain, taking into account their characteristics.

## Background

Prostate-Specific Antigen (PSA) screening for early detection of prostate cancer (PCa) is a much discussed topic. PCa is among the most common cancers and the sixth leading cause of cancer death among men worldwide [1]. In 2008, almost 1 million new PCa cases were identified and a quarter of a million deaths occurred globally [1]. PSA screening is controversial as the harms of population-based screening are liable to outweigh the benefits [2]. This is mainly due to over-diagnosis of clinically insignificant tumors that would never have affected the quality or quantity of a man’s life. Today, while there are numerous methods from nomograms to genomic tests to stratify the risk of PCa, it is impossible to definitively predict which PCa may progress over time, resulting in a high risk of overtreatment and associated irreversible adverse effects (e.g. urinary incontinence, erectile dysfunction, etc.) [3]. Furthermore, the PSA test sometimes misses significant PCa. The American Urological Association (AUA) and US Preventive Services Task Force (USPSTF) guidelines recommend that average-risk individuals aged 55–69 can be offered PSA after discussing benefits and harms with their clinician, while European guidelines, among others, recommend PSA screening in men aged > 50 years and men aged > 45 years if they have a PCa family history [4, 5].

**Fig. 1:**
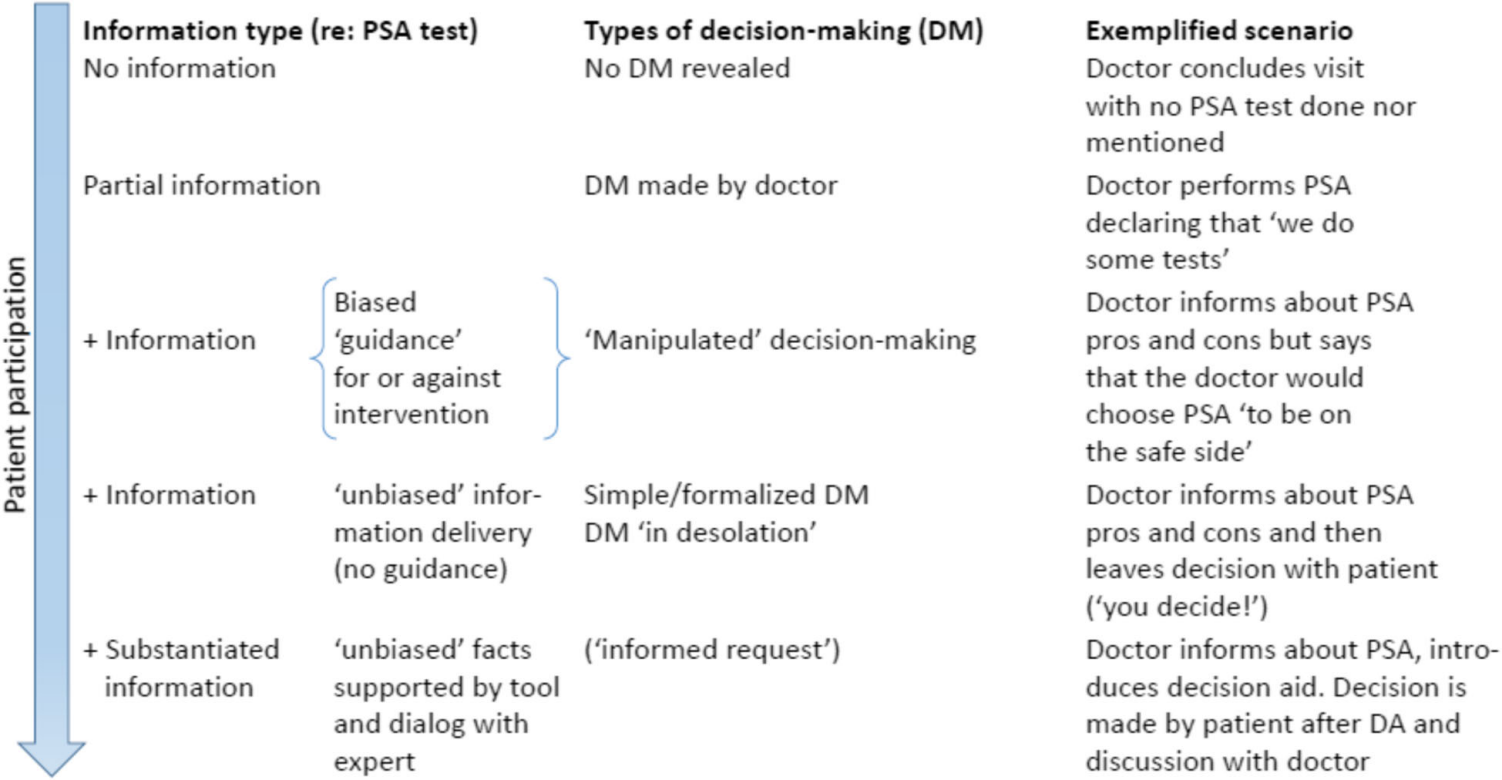
Spectrum of patient participation in PSA decision-making included in the survey

Some may think that the possible benefits outbalance the side effects associated with the test and ensuing treatment [2]. This decision can be difficult and may depend on characteristics of the individual, but also may depend on the information offered about PSA. The AUA and USPSTF therefore recommend Shared Decision Making (SDM) when deciding about having a PSA test [4]. In SDM, patients communicate with health care providers about possible treatments using the best available evidence while weighing treatment options and considering patients’ personal preferences [6]. It is advised that clinical situation-specific ‘*Decision Aids*’ (DAs) be used to assist to systematically and uniformly communicate risk information and highlight individual patient preferences without relying on clinicians’ memories which will often vary [6, 7]. DAs are used *in conjunction with* a dialogue with health care professionals when making *preference-sensitive decisions*, clinical situations where there are more than one reasonable testing or treatment option. Although guidelines support the use of SDM, the decision about conducting a PSA test, like most other health care decisions, are made across a spectrum of patient participation (see Fig. 1). In parallel, along this continuum, the amount of information delivery may vary.

The principle of patient participation in decision making is rooted in ethical considerations about autonomy and the individual’s right to self-determination regarding his or her own life [8]. Numerous considerations may, however, speak in favor of patient participation in choices about medical care. Among the rationales for sharing the process of making choices are the promotion of patients’ ownership of choices made, improved adherence, patient empowerment, and ensuring the right treatment for the right patient [6]. Additionally, through patient participation the responsibility for possible adverse outcomes may to a greater extent be shared between the clinician and the patient [9]. In this regard, research has suggested that poor communication and patients’ feeling that their perspectives have not been considered often is a major reason when patients decide to complain about health care [7, 10–12].

Many countries enforce patient participation principles through a legal requirement for obtaining the patient’s informed consent prior to any health care procedure as a modicum. Legislation and underlying principles about autonomy commonly assume that patients actually desire to receive pertinent information and want to be asked about their opinion. Nevertheless, some clinicians and researchers have doubted this assumption [13]. Even if ethical principles would advocate patient participation, only limited knowledge exists about patients’ own views on participation (e.g., in decisions about PSA) and to what degree their attitude depends on factors, such as age, personality, educational background, experiences with illness and health care, the possible consequences of decisions made, etc. Taken together, we have limited empirical knowledge about health care users’ preferences for participation and to what degree information about options and participation in decision-making affect satisfaction with health care. We know little about the significance of prior participation when treatment is unsuccessful and about whether participation may counteract a health care user’s dissatisfaction with care. Correspondingly, even if patient involvement may promote a patient’s identification with the choices made, empirical studies are called for to explore to what degree it may affect a patient’s likelihood to initiate a malpractice complaint [14]. The present knowledge gap may partly be due to difficulties with assessing such aspects of preferences and reaction patterns. One approach to examine preferences is to ask health care users about their views on different types of participation as exemplified in vignettes. In this regard, PSA screening provides a good model for studying men’s opinions regarding participation, as risks and benefits are finely balanced and a choice for or against using PSA potentially has many consequences.

In designing a Danish national survey using case vignette variants relating to the choice of PSA, we wanted to investigate the relative effect of different levels of participation on men’s satisfaction and readiness to initiate a malpractice complaint, while taking into account health care outcomes and health care user characteristics. To ensure that the survey was understandable to respondents and the case vignettes as true to “real-life” as possible, we chose to use a participatory design and to involve patient and public representatives in the development of the survey. Involving users in this kind of health care research may have many beneficial purposes that will be discussed later [15]. However, some immediate reasons deserve mention. Patient and public involvement (PPI) in research may ensure connection of study objectives and contents to the ‘real world’ and increase the chance that the survey will measure what we want to measure [15, 16]. Collaboration during the study preparation may serve as a first prerequisite for success [17]. Hence, feedback from a representative PPI group including cancer patients is crucial to ensure utilization of participant resources as efficiently and reasonably as possible.

The aim of this paper is to describe PPI in the development of our national, web-based case vignette survey for studying men’s view on participation in decision-making about PSA screening. As the paper describes PPI in our research project, Guidance for Reporting Involvement of Patients and Public (GRIPP) 2 principles have been followed [15]. The GRIPP principles are based on systematic reviews of the PPI evidence base and aim to improve the overall quality of PPI reporting. Briefly, this implies explaining the background and aim of PPI, describing the methods used for PPI, reporting on the results of PPI, commenting on PPI’s influence on the study, and critically reflecting on strengths and limitations of the approach taken [15].

## Methods

The purpose of our survey was to measure the effect of different levels of patient participation on satisfaction with health care and proclivity to initiate a malpractice complaint through requiring participating men to read through a constructed case vignette and respond to a questionnaire. The case vignette we used was designed in multiple variants with an identical core but differing with regard to the doctor’s information delivery to the patient, the choice made about performing a PSA, and the patient’s subsequent treatment and outcomes. This study design was chosen in order to cover the entire range of realistically occurring attitudes to patient participation (cf Fig. 1). Such attitudes include styles that strictly speaking may disagree with law requirements and therefore cannot be tested in real-life clinical encounters, but still occur.

For the survey, the age span 45–70 years was chosen, reflecting consideration of USPSTF and European recommendations [4, 5]. Respondents were asked to rate their imagined satisfaction with health care if subject to the situation illustrated in the vignette and assess the likelihood that they would make a formal complaint to the authorities about their health care. Additionally, respondents were asked to complete a questionnaire that assessed their general preferences with respect to participation in health care decision-making, their personal experiences with illness and health care, and some baseline information (e.g., age, marital status, level of education, and personality).

We build a web-based survey using Research Electronic Data Capture (REDCap) [18] and distributed personal survey invitations to respondents through the Danish authoritiy-provided digital mail box used for secure communication with citizens.

### Initial steps of survey development

The principal investigator (SB) together with members of the project group (SSP, MB, and NR) drafted a plan for the research project, vignette structure and choice of measurements that was finetuned through dialogue and feedback in the project group. The scenarios reflected the spectrum illustrated in Fig. 1 and were initially inspired by real life law cases described in the literature of patients who complained about their treatment [19], a previous case vignette survey [20], and a research paper about reactions of potential jurors to a hypothetical malpractice suit alleging failure to perform a PSA [21]. In order to fulfill the aim of the study, we constructed our draft vignettes as generic scenarios describing combinations of several levels of patient participation in health care decision making with different decisions regarding health care intervention (PSA), and various health care outcomes (see below).

An outline of the survey subsequently was agreed upon following a workshop with *academic* peers. In addition to 3 members of the project group (SB, SSP, and NR), the workshop participants were drawn from the medical psychology research group at the University of Southern Denmark (an assistant professor with expertise in quantitative research methods, an assistant professor with mixed methods background and experience with user involvement in research, and two research assistants with mixed methods backgrounds). The project group agreed on a preliminary survey draft with *30 different vignette scenarios*. The structure of the draft version is described in more details in Table 1.

**Table 1:** Content of draft version of survey following academic workshop

**Content of draft version of survey following academic workshop**	
• Brief description of the main study purpose	
• Questions regarding age, marital status, education, affiliation with labor market and experiences with illness and health care	
• ‘Control Preferences Scale’, a validated instrument that measured the degree of control an individual wants to assume when making decisions about medical treatment [22]^a^	
• Validated 10-item personality measure (the Big Five Inventory-10; BFI-10) [23]^a^	
• Subsequently, study participants were introduced to the case vignette set-up: “*Imagine that You yourself attend the following sequence of encounters with your doctor …*” . Participants were randomized into one of 30 different scenarios with an identical core structure though differing with respect to	
∘ (a) information from the doctor about the PSA test (five variants ranging from no information or participation of patients in decision-making to participation of patients through SDM; see Fig. 1)	
∘ (b) the decision to have a PSA or not	
∘ (c) three outcome scenarios (a favorable outcome with no PCa, a fatal scenario with non treatable PCa, and an intermediate scenario with PCa detected but succesfully treated) with reference to scenarios reported in the health law literature [19, 21]^b^	
• Following presentation of the case vignette, assessments were conducted concerning how satisfied the respondent would be with health care *if* he was the patient illustrated in the vignette and the likelihood that he would initiate a malpractice complaint. Items used for this assessment were modified from two previously validated instruments: The Patient Satisfaction Questionnaire, PSQ-18 [24] and the Consumer Complaint Intentions and Behavior questionnaire [25, 26]. Ratings were done on Likert scales (e.g. from very satisfied to very dissatisfied). Also participants were asked about their ability to identify with the patient in the scenario described in the vignette and their knowledge of the PSA test.	

^a^All English language instruments had been independently forward-backward translated by skilled Danish and English speaking translators following the procedures recommended by Beaton and colleagues [27]

^b^By way of example, in one version of the vignette, the fictional doctor performed the PSA test without information (‘*I will just take some routine tests*’) and the patient described in the vignette later was successfully treated for PCa. In another version, the patient chose not to have a test after being slightly nudged in disfavor of the PSA test and was afterwards diagnosed with a treatable PCa. In yet another version, the patient reviewed an SDM DA, after dialogue with the doctor chose not to take the PSA test, and was later diagnosed with a non treatable PCa. We used a DA translated and adapted from Burford, Kirby, and Austoker by Bro and Borre [28, 29]. The DA was publicly available on the Danish Health Authorities’ website (‘*PSA test for prostate cancer*’, da. [PSA-test for prostatakræft]; www.sundhed.dk)

### Patient and public involvement

For the further survey development, PPI representatives were involved in two steps: collaboration with a large group of adult men and with a small panel of patients with PCa experience.

#### Collaboration with large PPI group

Thirty men were engaged in the public sphere to assist with the development of the survey and case vignette versions. A ‘convenience’ sampling method was used by selecting men encountered at pedestrian streets, in market malls, in hospitals, and among academic course attenders with various backgrounds in Middelfart and Odense which are middle sized provincial towns in central Denmark. The number of men reflected the number of vignette variants agreed on following the academic workshop. PPI representatives were individually presented with a paper format draft of a single case vignette and supplementary questionnaire items. After PPI representatives had familiarized themselves with the draft vignette and filled out the questionnaire, they were questioned about their opinion of the study and the phrasing of vignettes, as well as their understanding of questionnaire items. Also, the time required to read through the draft vignette and complete the questionnaire was recorded. PPI representatives were encouraged to make suggestions to improve the case vignette and questionnaire items (wording and arrangement).

#### Collaboration with small PPI panel with PCa experience

Furthermore, collaboration with a three member panel of patients with particular PCa experience was arranged to further improve the questionnaire and the narratives illustrated in vignettes. Changes arising during feedback from the large PPI group would merit a new review with the panel. Likewise, in view of the survey’s focus on PSA and PCa, we found it reasonable to assemble a group with specific expertise in the patient perspective on PCa. Moreover, such a group might provide reflections on unseen research perspectives that might be considered particularly valuable from a clinical population’s point of view. The Danish Cancer Society served as a main entry to set up the panel. An introductory telephone meeting was coordinated with a regional PCa interest group through a main patient organization head. Following discussion with this representative about the survey, an initial in-person meeting was held with a team-leader of the regional interest group. After briefing the team-leader about the survey, a half day workshop was arranged. We arranged the PPI panel workshop with the team-leader and two other members of the regional interest group. Among the members, one was an academic with a PCa diagnosed at age 67 through PSA screening without prior symptoms. Another member was a blue-collar-worker diagnosed with PCa at age 56 after an elevated PSA following difficulties with urinating. The last member was an academic with a PCa diagnosed at age 67 after an elevated PSA following frequent urinating during the night. No one had active PCa. All agreed to be anonymously mentioned in e.g. research reports/papers. After a further introduction to the survey, its purpose and methods, there was a discussion about the focus of the project, the methods, and about research questions that might be particularly interesting from a PCa patient’s perspective.

## Results

### Collaboration with large PPI group

As no well defined measure of potential PPI collaborators could be established among e.g., passers-by in the mall, it is not possible to report on the ‘response rate’. Information on group composition is summarized in Table 2.

**Table 2:** Group composition in large group of PPI representatives (*N* = 30)

	Number	Median (range)
**Age in years**	27^a^	54 (24–71)
	**n**	**%**
**Marital status**		
Married / registered partnership	22	73
Living with a partner	2	7
Partner, not living together	2	7
No partner	4	13
**Highest education completed**		
Primary school	6	20
High school exam	2	7
Educated blue collar worker	8	27
Short-term higher education (< 3 year)	3	10
Middle-term higher education (≤ 4 years)	4	13
Longer-term higher education (> 4 years)	7	23
**Affiliation with labor market**		
Employed	22	73
Not employed	8	27
**On sick leave**		
Yes	1	3
No	29	97
PPI representative had had cancer himself last 5 years^b^	2	7

^a^ *n* = 27 as age not obtained in three men

^b^ One had had PCa

The time required to read through the vignette and respond to the questionnaire was measured in 23 individuals. On average, completion time was 11 min (median = 11; range 7–25 min).

After completing the paper survey, we discussed PPI representatives’ interpretation of the case vignette and the items. Emphasis was particularly on items that were difficult to understand. Generally, PPI representatives were accepting of the questionnaire and the case vignettes and after having looked through the survey, they usually became enthusiastic about the relevance of the survey and about providing feedback. Nevertheless, PPI representatives raised several issues (please see examples in Table 3).

**Table 3:** Main issues raised by PPI representatives

PPI representative suggestions		Comment	Consequence
Survey structure	Case vignette should be the very first part of the survey followed by questionnaire items	The rationale was that respondents may expect that the survey quickly gets to the subject matter and PPI representatives argued that the vignette was the central part of the survey	Case vignette moved to the first section of survey
	Alter the succession of questions following the scenario	For example grouping of vignette related items immediately following the vignette	Changed succession of items
Survey contents	General need for brevity of text and items whenever possible	PPI representatives maintained preference for minimum writing	Several adjustments with removal of unneeded text
	Need for a brief but comprehensible introduction to the hypothetical scenario	PPI representatives maintained importance of guidance throughout parts of survey	Insertion of brief prologues
	‘Reverse’ wording of questions	By way of example, PPI representatives suggested replacing ‘I would not use that doctor again’ with ‘I would use that doctor again’ because the original question may ‘push’ responders towards negative ratings	Rephrasing of items

When interviewed about the case scenario describing a lethal course of PCa, no respondents perceived it offensive or unethical. Specific questioning of a subgroup of PPI representatives (*n* = 8) revealed that all eight reported that they were able to identify with the patient’s situation described in the vignettes.

Moreover, responses to paper versions of the survey provided information regarding rating patterns. There was no indication of any ceiling effects (i.e., strongly skewed distribution of ratings to either side of the mean). Table 4 shows the allocation of responses to one of the key questionnaire items (participants’ imagined satisfaction with health care). Based on the rationale mentioned by PPI representatives (Table 3), subsequent discussion within the project group resulted in the decision to place the case vignette first. However, this structural change needed to be tested in the planned PPI panel. Additionally, regarding the items about complaint behavior, we found that a 5-point Likert scale (rather than 3- or 4-point ratings) was needed throughout the questionnaire to provide more information and stimulate participants’ reflection about responses.

**Table 4:** Distribution of PPI responses to a questionnaire item (*N* = 28)

	Very satisfied	Satisfied	Neither satisfied nor dissatisfied	Dissatis-fied	Very dis-satisfied
How would you define the level of satisfaction with the doctor’s care?	3	13	5	6	1
Percentage	11	46	18	21	4

### Collaboration with small PPI panel with PCa experience

Feedback obtained from the larger group of PPI representatives during the process described above resulted in a number of changes that merited reconsideration of the survey (moving of vignette; shortening and simplification of text passages; improved introductions to the case vignette and questionnaire sections; reversal of particular questions; and use of a 5-point Likert scale). Subsequently, we went through the survey structure with panel members. Again we checked comprehension of items from the lay perspective and also re-examined the contents of invitation letters. An interactive and informal format was applied to facilitate exchange of thoughts and enhance discussion about paper and draft web versions. Panel members specifically highlighted a number of themes, please see Table 5.

**Table 5:** Main issues raised by PCa panel members

PCa panel member suggestions		Comment	Consequence
Survey structure	Feedback from user panel regarding optimized position of item	The user panel members recommended to move the question about whether the respondent could identify with the patient in the case vignette	Item rearrangement
Survey contents	Necessity of further introduction	Panel members reiterated the wish for PPIs for introductory explanation. For example “*Now we ask you some questions regarding your personality*”	Further information inserted
	Need for clarification	Emphasize that the doctor described in the case vignette is the fictional patient’s general practitioner	Clarification of vignette contents
	Omission of superfluous vignette information	For example omission of “*repeated prostate biopsies verifies*” from PCa scenario	Case vignette text reduction
	Change of wording	E.g. ‘tell others about experience’ instead of ‘badmouthing’	Rewording item
	Replacement of technical terms	For example replacement of ‘screen’ with ‘test all’	Rewording item
	Eradicate a ‘don’t know’ category	Deletion from socio-demography baseline information question	Item adjustment

Among additional themes, a panel member noted that there were two questions in one of the items of the 10 item personality instrument (“*I see myself as someone who is relaxed, handles stress well*” [23]). Likewise, it was recommended by panel members to re-consider whether ‘recurrent contact with health care sector’ should be deleted or retained among socio-demography baseline information questions. The acceptability of fatal scenarios were discussed from a cancer patient’s perspective. Panel members generally found case descriptions acceptable (as fatal scenarios ‘just illustrate the sad reality of some cancers’). There was a broader discussion about the project, which panel members found important. Some research questions were deemed of particular interest from a patient perspective, including the impact of various socio-demographic, personality, and other factors on preferences for having a PSA test for PCa.

The project group discussed the PPI panel comments and generally found the recommendations worth following. A few points, however, remained unchanged. For example, although interpretation may vary, the project group considered it reasonable to keep the ‘recurrent contact with health care sector’ item (see above) and to retain the ‘double-question’ personality item mentioned above as a part of a standardized and validated instrument. Following the changes to the web-version of the survey, it was pilot-tested by panel members. Afterwards, we made some further small adjustments mostly related to layout. Panel members received no remuneration apart from a paid lunch during the workshop and two bottles of red wine to take home.

Passages from one of the case vignette versions are shown in Table 6.

**Table 6:** Extracts from one case vignette version

“*Your doctor tells you about a blood test for prostate cancer. It is called PSA. PSA is a natural enzyme produced by the male prostate gland that can be measured in blood. The test is used for diagnostics and control of prostate cancer treatment. PSA blood levels normally increase with age, prostate gland enlargement, and if the prostate is sick (*e.g.*, cancer). However, an increased PSA does not necessarily mean that you have prostate cancer. The doctor then tells you that ‘it is not common’ to test all for prostate cancer with PSA because the test is not good enough. One can for example have increased PSA in the blood without having prostate cancer. In addition, prostate cancer may develop slowly so that you experience no prostate cancer symptoms before dying from other causes. Furthermore, the treatment of prostate cancer may have significant side effects.* ***You decide to have a PSA test done***”	
“*It appears that you have prostate cancer. During the course of treatment, you and your family get increasingly worried and see your doctor several times. Fortunately, it is possible to remove the cancer by surgery without any further complications* […]”	

## Discussion

In this paper, we describe PPI in the construction of a national, web-based case vignette survey for studying men’s view on their participation in decision-making about PSA screening.

Involving relevant stakeholders, including PPI representatives, in the development of research projects has been repeatedly shown to ensure that, e.g., the survey to be deployed is relevant, functional and acceptable [15, 17]. Therefore, we chose to employ a health care user-centered design and an iterative process throughout different phases of the survey development. We require future survey participants to both respond to questions about real life facts as well as hypothetical questions.

The approach of asking participants to familiarize themselves with imagined patient scenarios necessitates thorough exploration into what would be common interpretations of constituents of the vignette variants. In this regard, PPI proved very valuable. Repeated feedback from PPI representatives during stages of the survey development ensured a continuous grounding in *users’ understanding* and allowed for a gradual improvement of vignette scenarios and questionnaire items. Representatives simultaneously provided the project group with a greater insight into the issue under study and with a better rapport with the community by expressing perspectives that reflected different but first-hand knowledge [30]. Unsurprisingly, this was especially the case with the patient panel. By way of a simple example, we could delete considerable amounts of superfluous information that we had believed to be important to a layperson. Likewise, representatives repeatedly identified an unfortunate overtone of draft questionnaire items that could lead study participants to respond unintentionally negatively. As a result, we realized the necessity for reversing the formulation of items derived from previously validated instruments [25, 31]. More in-depth analyses, critical reflections, and discussion gradually came up and verified that spending sufficient time is a critical factor in PPI [16, 30]. It is crucial to spend sufficient time with PPI members in order to build a good rapport, so that they feel sufficiently safe to challenge potential preconceived assumptions of researchers about the material under development [30].

Overall, through working with PPI representatives, we aimed to increase response rates of our future survey by keeping it as short as possible, adjusting language to meet the user perspective, and by including information about estimated time to complete the survey in the invitation material [32]. Besides, PPI served to warrant the survey’s *acceptability* among potential respondents. Respect for other people’s time and well-being itself demands prior discussion with representatives of the target population before launching a large-scale survey. The latter pertains not least to a survey that illustrates scenarios on cancer management with fatal outcomes that may cause emotional distress and where some study participants may be real life cancer patients.

Dependent on the time of involvement, lay people also may help prioritize research topics, thereby offering important input into research plans that sometimes involve very abstract ideas [15, 33, 34]. In our project, collaboration with the patient panel naturally provided a format especially suitable for discussing patient relevant research questions. In this regard, it is noteworthy that panel members perceived variability in patient preferences regarding use of medical tests to be a particularly important issue.

Apart from requiring some more time and research team resources, we identified no negative effects from PPI in our research design. It was our impression that following engagement, PPI representatives drawn from the public setting as well as from the patient organization became very committed. In this regard, it is noteworthy that although time spent on participation is important for PPI members regardless of how long it takes, the first group received no means of remuneration [16, 34]. Anyway, we considered that participation demanded relatively little time and offering reimbursement of any kind would appear cumbersome and inconvenient. For the patient panel, more time was required, and it felt natural to offer a small gift as well as meals during the workshop.

### Limitations

In research with PPI, apart from explaining its purpose, providing a description of methods used for involvement, and its results, GRIPP 2 principles recommend critical reflection on what could be improved [15]. Considerable steps in designing our research project had been taken before PPI was undertaken. Our study was driven by a conceptual interest into how participation of health care users in decisions about PSA may influence their perception of health care. From the beginning, this greatly influenced our study scope and we did not involve patients or the public in these stages of our survey development. By way of an important example, asking for PPI comments on research questions that are largely pre-defined by the project group may seem a little backwards. In part, though being a poor substitute for the patient voice, we attempted to account for this through initially obtaining ‘third party’ feedback and advice during the academic workshop [16].

When involving users in research, it is recommended that diverse groups are reasonably represented [16]. We aimed to ensure diversity regarding age and socioeconomic background in the *large group* of PPI representatives by recruiting men from various settings and our large group PPI composition suggests some heterogeneity was achieved (see Table 2). Collaboration, however, necessitates that potential users want to participate. Some men chose not to participate, although they could potentially have provided particularly relevant feedback. Ensuring participants with a broad range of health literacy in a study regarding preferences for involvement in health care decision making might be important, as health literacy might influence their impressions of and suggestions for survey development. In our study, we did not assess PPIs’ levels of health literacy. However, we sought to ensure that various educational backgrounds were represented. Even though this was succesful in the large group of PPI representatives, diversity was less in the *small patient panel* with two members being academics. To our knowledge, no PPI participant had severe, incurable cancer. Involving such men might be difficult, however, lacking their perspective could be problematic, as we have little knowledge about their perception of the fatal scenarios of the survey. We tried to partly account for this by particularly discussing these scenarios with panel members who themselves had gone through the distress associated with cancer, well knowing that they felt they had been successfully treated.

It is possible that more men could have participated in the large PPI group to have two or more members review every one of the 30 vignette variants. Our approach reflects a compromise. Then again, it must be kept in mind that vignette components are identical across scenario combinations. As a result, three participants reviewed each of 10 variations in vignette descriptions of patient participation, and 10 participants reviewed each of three possible outcome types.

Some limitations regarding PPI representatives’ feedback on the hypothetical questionnaire scenarios deserve mention. Comments during survey development indicated that they could identify with the patients in the situations described in vignettes, but some points must be kept in mind. Case vignettes have been previously used in studies of health care decision-making in prostate cancer [21, 35], however, it is important to emphasize that participant responses to vignettes reflect *hypothetical judgements* and not real-life behavior. Hence, we cannot rule out that responses might have been different in a factual situation. It can be very difficult to relate truly realistically to a critical situation that one has never experienced. In the context of discrete-choice experiments, it has been argued that patients’ preferences for choosing hypothetical scenarios may differ from their preferences for making actual treatment decisions (‘hypothetical bias’) [36]. This caveat seems contradicted in a few studies comparing actual choices with stated preferences [36, 37]. Again, it should be noted that studies mostly have looked at decision-making from the health provider perspective. Overall, there is a scarcity of research concerning the external validity of methodologies applying hypothetical choices [38]. Furthermore, in our survey, respondents are passive witnesses to the course of health care described without any personal influence on the decision-making. Respondents only influence their judgement of health care as expressed in the attached questionnaire. Alternatively, respondents could have been granted the possibility to ‘participate’ in the mock up scenario e.g. at the step of deciding whether to have the PSA test done. Constructing the survey in such a way that it is possible for the respondents to decide whether to have the PSA test done might be an attractive approach in future studies. However, this would require additional steps to ensure the necessary number of respondents for each of the 30 different scenarios and achieve a satisfactory statistical power. Another potential limitation of our study is the failure to include women. As women may have an influence on men’s health behaviours and health care choices, it may be important to know what the partners of men think about participation in decision-making. However, including only men in our study reflects a decision to sharply focus on health care users’ perspectives regarding their own healthcare matters.

## Conclusion

There are many arguments in favor of involving health care users in decisions about health care and clinical guidelines recommend that patients and clinicians make choices about having a PSA through SDM [4]. However, we have limited empirical knowledge about the view of various groups of health care users on participation in decision-making. Likewise, we know little about how participation may affect satisfaction with health care or the risk of malpractice complaints and how to best study such effects. In this paper, we describe PPI in developing a survey that aims to measure the association between various approaches to participation and men’s satisfaction with health care and readiness to complain, while taking into account health care outcome and participant characteristics. Although the survey design described has its limitations, we consider it a necessary step towards getting a deeper understanding of patient participation and its potential to promote satisfaction and prevent malpractice litigation. The method suggested may serve as a model approach to future studies on the cutting edge between patient desires regarding participation, satisfaction with health care, and the legal aspects of patient participation. Even though our success with PPI remains to be fully determined through real life implementation of the survey, our experiences point to the desirability of using PPI in this kind of research.

## Data Availability

Not applicable.

## Abbreviations

AUA: American Urological Association
BFI: Big five inventory
DA: Decision aids
GRIPP: Guidance for reporting involvement of patients and public
PCa: Prostate cancer
PPI: Patient and public involvement
PSA: Prostate-specific antigen (PSA)
PSQ: Patient satisfaction questionnaire
REDCap: Research electronic data capture
SDM: Shared decision making
USPSTF: US Preventive Services Task Force
